# What is the role of global health and sustainable development in Swedish medical education? A qualitative study of key stakeholders’ perspectives

**DOI:** 10.1186/s12909-023-04502-y

**Published:** 2023-07-17

**Authors:** Lotta Velin, Pia Svensson, Tobias Alfvén, Anette Agardh

**Affiliations:** 1 Centre for Teaching & Research in Disaster Medicine and Traumatology (KMC), Department of Biomedical and Clinical Sciences, Linköping University, Johannes Magnus Väg 11, Linköping, 583 30 Sweden; 2 Social Medicine and Global Health, Department of Clinical Sciences, Lund University, Malmö, Sweden; 3 Department of Global Public Health, Karolinska University, Stockholm, Sweden; 4 Sachs’ Children and Youth Hospital, Stockholm, Sweden

**Keywords:** Medical education, Global health, Sustainable development, Curriculum development, Qualitative research, Interviews, Sweden, Sustainable development goals, UN 2030 Agenda

## Abstract

**Background:**

Global health and sustainable development have increasingly been recognised as important parts of medical education, yet education on these issues remains fragmented and scarce. In 2020, a bill to reform the national medical curricula across all Swedish medical schools was introduced, including a greater emphasis on global health and sustainable development. This study aimed to explore the perspectives of key stakeholders in medical education on the role of global health and sustainable development in Swedish medical education.

**Methods:**

This was a qualitative study based on semi-structured interviews with 11 key stakeholders in medical education, broadly defined as faculty board members (dean and/or vice-deans for medical education) and/or programme chairs representing six universities. Data were analysed using qualitative content analyis (QCA). The study was conducted according to the Consolidated Criteria for Reporting Qualitative research (COREQ) guidelines.

**Results:**

Stakeholders discussed the challenges and opportunities associated with the modification of medical education, which was seen as necessary modernisation to fit the changing societal perception of the role of medical doctors. The anchoring process of redesigning the curriculum and integrating global health and sustainable development was discussed, with emphasis on ownership and mandate and the role of teachers and students in the process. Finding a shared understanding of global health and sustainable development was perceived as a challenge, associated with resistance due to fear of curriculum overload. To overcome this, integrating global health and sustainable development with other topics and developing existing components of the curricula were seen as important. Additionally, it was stressed that fostering capacity building and developing infrastructure, including utilization of digital tools and collaborations, were essential to ensure successful implementation.

**Conclusions:**

Medical institutions should prepare future doctors to respond to the needs of a globalised world, which include knowledge of global health and sustainable development. However, conceptual uncertainties and questions about ownership remain among key stakeholders in medical education. Yet, key stakeholders also highlight that the inclusion of global health and sustainable development in the new curricula represents multiple overarching educational opportunities that can bring about necessary improvement.

## Background

Global health has increasingly been recognised as an important part of medical education, in part due to the growing awareness of the importance of globalisation for health and health systems. Global factors such as international migration, climate change and transmission of pathogens across national borders, as exemplified by the Covid-19 pandemic, require healthcare practitioners to have an understanding of such phenomena and the interconnected nature of health with the world in which we live. Healthcare practitioners have reported significant benefits of global health experiences in their clinical practice [1], and researchers have called for more global health opportunities in medical school to equip future medical professionals to face an increasingly interconnected world [2, 3]. Although global health has previously often been understood as “tropical medicine”, more recent conceptualisations focus on achieving health equity, with particular focus on marginalised communities [4–6]. Additionally, modern definitions of global health emphasise a holistic approach, inlcuding emphasis on social determinants and “root causes” of health and illness, thereby recognising the interconnectedness of health with social, economic, environmental, and political factors [7, 8]. The field of global health is closely related to and often discussed in relation to sustainable development, where the United Nations’ declaration on the 2030 Agenda is central [9]. The Agenda outlines one “health goal”, although all 17 goals for sustainable development can be linked to health and health determinants [10].

Previous studies in a European context and elsewhere have shown that medical students have a strong interest in global health [11]. However, although global health education is often available solely through elective courses [12], students have suggested that integration in the core curriculum would be preferable to ensure that all medical students receive such education [13]. In a previous German survey, medical students and educators identified the most important barrier to global health education to be the low priority given to global health by faculty members and academic management levels [14]. The second major obstacle was the lack of institutional structures and support [14]. In a qualitative study conducted in Canada, global health academics similarly expressed frustration at the existing fragmentation and the lack of strategic direction, financial support and recognition from the university [15].

According to a study from 2015, global health has not been taught in all medical schools in Sweden, and it was stated that most students completed their medical degree without any global health education [16]. In 2013, a proposal for a new medical programme in Sweden was set forth, which included a greater emphasis on *“global perspectives”* and *“social responsibility”* and learning outcomes on health systems globally and nationally [17]. This proposal also stated that *“the future role as a doctor is also affected by the increasing global mobility of patients, populations and health and medical services staff”* and specifically stated learning outcomes relating to global health and sustainable development [17]. In 2020, this proposal was agreed upon, and all seven Swedish medical schools had begun the transition to the new programme by the end of 2021.

With implementation of the new Swedish medical programme underway and the growing interest for integration of global health and sustainable development in medical education across Europe, the role of global health and sustainable development in medical education is a timely issue [17]. Faculty and programme leadership have key roles in the development of medical curricula, but to our knowledge, no study has yet explored their perspectives on global health or sustainable development. Therefore, this study aimed to explore the perspectives of key stakeholders in medical education on the role of global health and sustainable development in Swedish medical education.

## Methods

### Study design

This was a qualitative study based on semi-structured, in-depth individual interviews and manifest and latent qualitative content analysis (QCA), according to Graneheim and Lundman [18]. QCA is suitable for exploring different perspectives and participants’ lived experiences of a phenomenon. As such, it emphasizes variations within the text to attain a broad description of the phenomenon in question.

See below for further details. The study was conducted according to the Consolidated Criteria for Reporting Qualitative research (COREQ) guidelines [19].

### The Swedish medical programme

The new programme is a six-year programme (compared to the previous programme which was five and a half years) and leads directly to a medical license, whereas the previous programme led to a M.D. but required a minimum of 18 months of standardised clinical service as a “medical intern” to obtain a license to practice independently. Although the structure of the medical programme varies in the different universities, typically the first two or two and a half years focus on pre-clinical teaching, whereas the remainder of the programme focuses on clinical education, including clinical rotations. Each one of Sweden’s seven universities with a medical programme has been asked to design new curricula, in accordance with the national proposal.

### Sampling and recruitment

Since different universities have different faculty and programme structures, *“key stakeholders”* in medical education were broadly defined as faculty board members (dean and/or vice-deans for medical education) and/or MD programme chairs. Potential participants were identified using a purposive sampling method, based on the criteria of being a *“key stakeholder”* in one of Sweden’s seven medical programmes. University websites with information about the faculty and programme leadership were reviewed. All eligible participants (ranging from 1 to 3 per institute) were invited to the study through an email including a standardised study invitation text, and all participants who did not respond were sent a reminder. Of the fourteen eligible participants contacted, eleven agreed to participate, representing six universities.

### Data collection

Interviews were conducted through video-calls via Zoom [20], using a semi-structured interview guide with thematic areas focusing on global health and sustainable development. The interview guide consisted of open-ended questions, and probes were used to encourage participants to further elaborate on their responses. Two versions of the interview guide were developed, with one targeting participants at the faculty level and one intended for participants at the programme level. The two versions had minor differences in wording adapted to the differing roles and covered the same thematic areas. The first two interviews of study participants (faculty level, programme level, respectively) served as a pilot testing of the interview guides. The pilot interviews were included in the analysis as only minor adjustments were made to the interview guide. The interviews were conducted by the first author, who was a medical student at the time of the interviews. The interviewer had no relationships with interviewees prior to the study commencement.

All interviews were conducted in Swedish. The duration of the interviews ranged from 17 to 34 min. The audio recordings were transcribed by the first author ad verbatim together with field notes. Data collection took place between June 1st – September 11th 2020.

### Data analysis

Data were analysed using qualitative content analyis (QCA), by following the steps described by Graneheim and Lundman [18] and managed with Nvivo software, version 1.6.2 [16]. The two sets of interview transcripts (faculty level and programme level) were merged for the purpose of analysis. The transcripts were read carefully by all authors to gain familiarity with the data. This was followed by a procedure of decontextualisation, whereby the transcript texts were divided into meaning units that were assigned codes. The coding was initially performed by two of the authors separately (PS and LV) and then conjointly to identify discrepancies and reach consensus. Through an iterative process of moving back and forth between the codes and the transcripts, three of the authors (LV, PS, AA) examined the coded data for similarities and differences between codes. Codes were recontextualised and clustered into sub-categories, and then grouped under broader categories. Any disagreements were resolved through discussions. The sub-categories illustrated the manifest level of analysis, highlighting the voices of the participants, while the categories represented the latent interpretations of the collected data.

### Ethical considerations

Prior to the interviews, all participants were given written and oral information about the study. They were informed that their participation was voluntary and confidential and that they had the right to withdraw at any time point. All participants were given the opportunity to ask questions about the study, before providing oral consent. No ethical approval was needed for this study according to Swedish legislation, as no sensitive information was collected. All results were presented in a anonymised manner to ensure confidentiality.

## Results

**Fig. 1:**
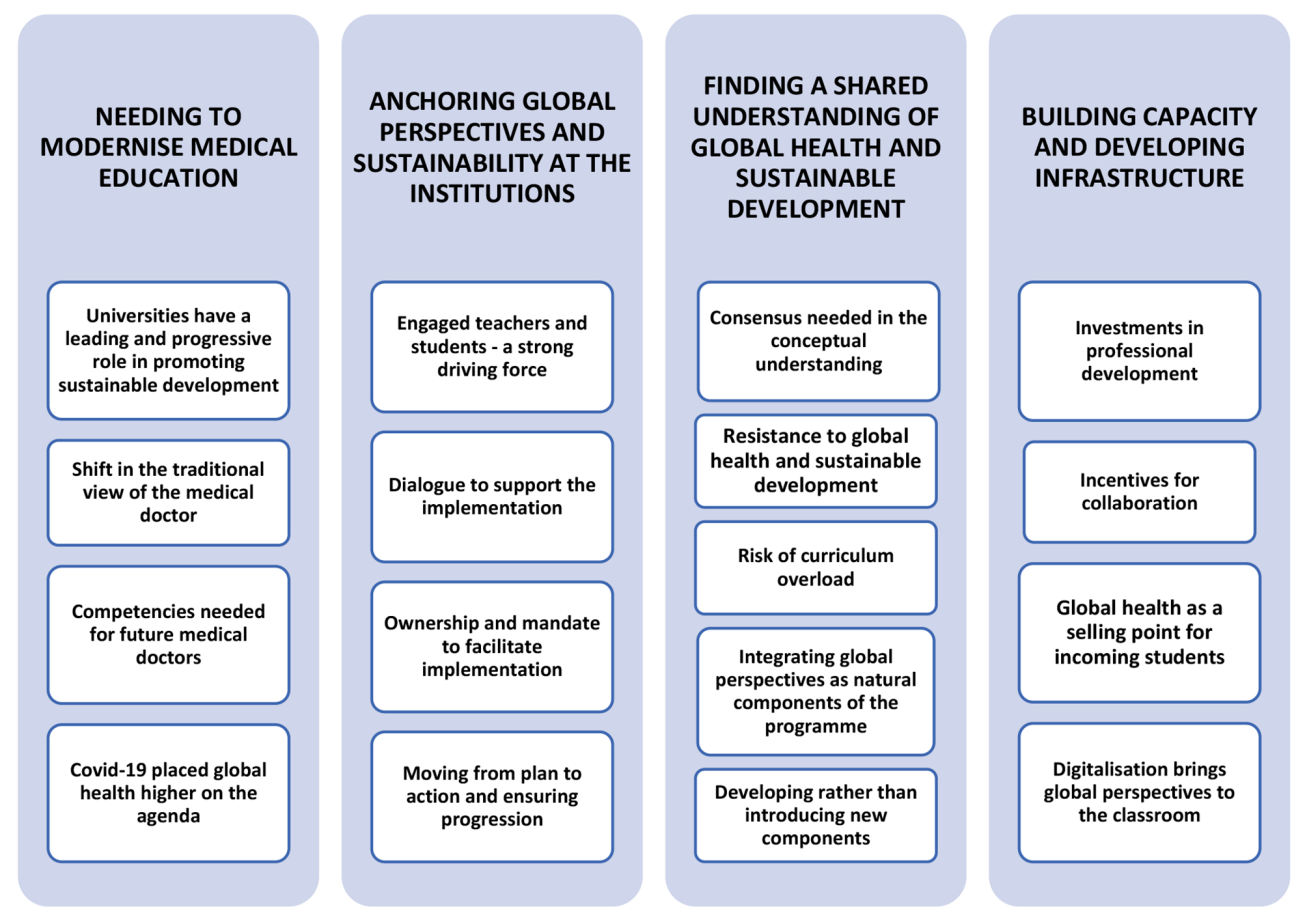
Categories and sub-categories emerging from the analysis

This study is based on interviews with eleven key stakeholders (three men and eight women) within the medical education in Sweden. Seven of the participants represent programme directors, and four represent faculty members. An overview of the final analytical model is shown in Fig. 1, consisting of seventeen sub-categories and four categories.

The results are presented in the text showing categories as headings and sub-categories in italics. IP under the quotes indicates the Interview Person.

### Needing to modernise medical education

#### Universities have a leading and progressive role in promoting sustainable development

Reflecting on the integration of global health and sustainable development in the medical curricula, participants highlighted universities’ leading and progressive role in the work for sustainable development. They expressed the view that universities are powerful stakeholders with the potential to influence society. Furthermore, they argued that educational institutions have a responsibility to lead by example in matters that concern societal development.

*“What we do shapes what others do, so it is important that we lead by example…if we show that we take these questions seriously, so will others.” (IP10, Faculty board)*

#### Shift in the traditional view of the medical doctor

The participants argued that the traditional view of the medical doctor has changed from mainly focusing on curing diseases to an increased attention on their role in primary prevention and leadership for public health. Both programme directors and faculty board members believed that this shift must be reflected in the medical programme and that medical education needs to prepare students to be able to take leading and decision-making roles in the society, beyond clinical practice.

*“In the role as a doctor it is not unusual that you will end up in some kind of leading position, and then you need this knowledge, and set an example to work in that direction.” (IP2, Faculty board)*

According to the participants, since medical education has its origins in a traditionally conservative discipline, aspects related to global health and sustainable development have not previously been prioritised. The reform of the medical education through the new six-year medical programme was therefore seen as an opening for new opportunities to modernise medical education, among other ways by including global perspectives in the curriculum.

*“Now we have a perfect opportunity to actually change an education.” (IP3, Faculty board)*

#### Competencies needed for future medical doctors

Participants stressed that medical students must be fostered in critical thinking regarding social determinants of health, health equity and the right to health. Furthermore, they stated that it is critical that the programme reflects the society we live in and prepares future medical doctors to encounter future needs. Globalisation and increasingly heterogeneous and multicultural societies require doctors to have knowledge about transnational transmission of infectious diseases and skills to encounter patients with various demographical profiles and cultural backgrounds.

*“I think it´s a precondition to be a doctor in a modern world. To be able to encounter all kinds of people and provide equal treatment. I think you must have a global health perspective with you as a modern doctor.” (IP10, Faculty board)*

#### Covid-19 placed global health higher on the agenda

The global spread of Covid-19 had contributed to placing global health higher on the agenda at the educational institutions. It had led to an increased awareness of and interest in global perspectives and the need for preparedness related to pandemic outbreaks.

*“There has never been as much focus on global health as there is now… perhaps the global situation and the world we live in now contribute to global health getting a completely new focus from the students… but also from us who are teachers and leaders of the organisation. I guess it is the time of opportunity.” (IP 10, Faculty board)*

### Anchoring global perspectives and sustainability at the institutions

#### Engaged teachers and students – a strong driving force

Commitment among teachers who are passionate about the issues was described as *“the most important driving force”*, a critical factor for the implementation and a potential source of inspiration to other teachers. However, building on this enthusiasm was still seen as a challenge.

*“There are global health enthusiasts [among the teachers], but integrating it in the system is hard.” (IP4, PD)*

Participants also perceived a strong demand from students to include more aspects of global health in the programme. Students were described as a positive force often involved in driving these issues, and several participants noted that many students have a strong interest in global health. However, it was also noted that there may be a bias, in that the students involved in the curriculum design may be more interested in these issues than the general student body. Student involvement in various working groups had been highly valued and it was felt to be particularly beneficial to increase student influence to understand what was missing in the existing curriculum.

*“We should always listen to the students, because as I say, you are the new generation who has knowledge about things that we don’t, because you are a product of the time you live in. Increasing student influence – I think this is really important, to understand what we are missing in the curriculum. What it is that you feel you are not getting. Here come young people with a lot of energy, and it is important to not lose it along the way. I think we do a poor job here sometimes – the energy gets lost.” (IP3, Faculty board)*

#### Dialogue to support the implementation

Participants shared different experiences of the implementation process, where some had to a larger extent engaged in dialogue and information activities concerning the integration of global health and sustainable development. Much effort was invested in convincing teachers and others about the importance of these perspectives and to recognise the relevance of global health and sustainable development in relation to their respective specialisations. The strategic approach also differed, and the universities varied in the extent to which they had appointed committees, working groups and process leaders to develop the curriculum and support implementation.

*“It has been an enormous amount of ground work to develop the curriculum, and I think that it has been a very good anchoring process... there have been a lot of activities targeting different course leaders as well as the entire staff…there have been numerous meetings and information to get everyone onboard.” (IP2, Faculty board)*

#### Ownership and mandate to facilitate implementation

Participants emphasised the importance of ownership and clear directions from leadership levels to find a common ground and mitigate resistance. Support at higher levels was also needed to ensure necessary investments in capacity building to accompany curriculum development. It was also stressed that the process would be easier if it was coordinated through a central function, with someone responsible for monitoring the implementation. Without ownership there was a risk that everyone perceived this as *“someone else’s responsibility”.*

*“The university needs to have a clearly defined function in this work and a clear structure for how teachers and programmes are to be supported in approaching these issues…the overall responsibility must lie at university level, and there needs to be a central function that handles these questions.” (IP11, Faculty board)*

However, some participants asserted that the discussion about the 2030 Agenda was more active on higher levels in the organisation and concerns were raised that there was a dissonance between senior leadership and the teachers, where some teachers experienced these issues as more peripheral.

*“The higher up in the organisation you get, if you look at the faculty board, the management, or if you look at the university board, these are active questions. The further down you get in the organisation, it is less visible, aside from the institution and teachers who are specialised in the topic.” (IP7, PD)*

#### Moving from plan to action and ensuring progression

Participants highlighted that the greatest challenge was to move from plan to action, to put the new course plans into practice and to sustain engagement among those involved. It was difficult to know if they had found a good balance, if there was a clear progression throughout the programme, if the teachers had the right competence, and if they had found an appropriate way of examining these topics.

*“The examinations will be a big challenge, to find a good way to do this. We have many dedicated teachers, but it takes time and money… especially to be able to capture the progression throughout the programme[…] they have not examined global health before.” (IP8, PD)*

### Finding a shared understanding of global health and sustainable development

#### Consensus needed in the conceptual understanding

According to the participants, challenges with implementation were partly due to how global health and sustainable development were defined, understood and communicated. Global health and sustainable development were often seen as broad and complex concepts related to social, economic and environmental issues, although some stereotypically associated global health with tropical diseases in *“developing countries”.* Participants were sometimes struggling to operationalise these concepts into tangible activities applicable in a clinical context. It appeared that there was a need to reach consensus in the conceptual understanding of the topics and more specifically, what it means in relation to medical education. Some participants stated that they awaited more concrete definitions and instructions before this work could be prioritised.

*“I think that there is an uncertainty in that sustainable development is such an enormous field. Where shall we put the focus? How much of it should we have in each course? It is a big challenge since we always have extensive amount of content to include in all courses. You need to make certain demarcations… I think many are concerned about this… (IP11, Faculty board)*

*“Everything is related to global health, but in order to apply it, get something done and move things forward it needs to be concretised - what are we talking about? By applying more concrete goals such as how we work with women’s health, children’s health, interpersonal violence, it will be easier to apply.” (IP9, PD)*

Lack of consensus within the faculty board was also explained by the fact that some key stakeholders viewed the 2030 Agenda as a political agenda and considered it to be inappropriate for state-run education institutions.

*“There is no consensus about sustainable development within the faculty board, but that’s because of how it has been presented… It has become a political issue, and the 2030 Agenda is seen as a political agenda. That’s the conflict ….We need to talk about this and most of all we need to de-dramatise it.” (IP3, Faculty board)*

#### Resistance to global health and sustainable development

Participants shared multiple challenges to the inclusion of global health and sustainable development in the curriculum, including the lengthy process that changing a curriculum entails and difficulties in striking a balance in the appropriate extent to which elements outside the clinical domain should be brought into the medical curriculum. Several participants described personal views, or views expressed by teaching staff, that global health and sustainable development were “*niche*” topics, with only basic knowledge needed by the majority of the students. In the interviews, it emerged that faculty/programme board members and/or teachers were frequently resisting efforts to expand the role of global health and sustainable development in the curriculum. This resistance was thought to be partly rooted in a perceived distance between global health and sustainable development and other scientific disciplines and traditions.

*“There is a great inertia within many clinical specialties to add a societal perspective.” (IP4, PD)*

Challenges at the level of the teachers were stressed, with considerable heterogeneity in attitudes and previous knowledge about global health and sustainability.

*“There are many different wills…you know, in one semester there are 60 different teachers… It’s not just to go out and talk to two or three persons, then it would have been easier. There are many people involved here.” (IP5, PD).*

#### Risk of curriculum overload

The perceived conflict between scientific disciplines and the *“softer”* topics of global health and sustainable development caused challenges for the implementation. It was often perceived *as “yet another perspective”*, “*a question among many other important questions in the medical programme”* or *“something that is already taken into consideration”* in an already dense programme and heavy workload. Faculty members and programme directors dreaded a risk of curriculum overload. Global health and sustainability were regarded as important topics, but not more important than other subjects of greater clinical relevance for medical students.

*“The challenge that we have all the time in medical education is to fit everything into the programme. There are so many things that you would like to include…” (IP10, faculty board)*

*“I think there is a perception of lack of time and resources…both in relation to developing a new curriculum for the medical programme and feeling overwhelmed by everything that should be included.“ (IP4, PD)*

#### Integrating global perspectives as natural components of the programme

To convey a holistic understanding, avoid curriculum overload, and facilitate the implementation, participants agreed that these perspectives needed to be integrated as natural components in the programme, not as a sidetrack. They underlined that they need to be applicable in a clinical context so that students and teachers can see the relevance to their professional development and particular field. Furthermore, the need to ensure progression of learning throughout the curriculum was emphasised, to foster deeper conceptual understanding and critical awareness over time. One suggestion was to include the perspectives in the clinical cases that the students work with throughout the education.

*“It cannot be something outside, it cannot be something called “global health”, it needs to be integrated throughout the entire education.” (IP3, Faculty board)*

Another strategy was to link the work with global health and sustainability with the universities’ ongoing work with internationalisation, which universities are obliged to work with.

*“It relates to the strategy for internationalisation that we are required to work with by law. This means that we must work with international issues during the education, and an action plan has been drawn up… here global health will be relevant in some way.” (IP1, PD)*

#### Developing rather than introducing new components

The approach for integrating global health and sustainable development in the new curriculum was to develop existing teaching materials rather than introduce new components. The strategy was to start with a thorough mapping of the courses to identify elements related to the 2030 Agenda and global health that are already in the programme, but which can be clarified, defined and further developed. This mapping process would also serve the purpose of detecting entry points in the existing curricula where these perspectives could be integrated, for example, in clinical cases.

*“I think that the important thing is not to view it as another thing to include the programme…that always causes problems, like “oh no are we going to do this also…” but no, you are already doing it, you just need to clarify it, you can develop the teaching, you can problematise it more from a global perspective.” (IP3, Faculty board)*

*“We need to go through the new course plans and see how these aspects are covered, if we need to create new components, or if we are already doing things that we just need to understand that we do.” (IP2, Faculty board)*

### Building capacity and developing infrastructure

#### Investments in professional development

To successfully develop the new curriculum and strategically organise the implementation, participants recognised the importance of investing in professional development and mobilising financial and human resources. Primarily, course leaders and teachers needed more knowledge on the topics of global health and sustainable development and guidance on how to integrate relevant aspects in their teaching and examinations.

*“There are many things that the teachers need training in now with the new medical programme...it is important not to set the goals too high... so that it is realistic to work with it.” (IP4, PD)*

*“There are still many teachers that need to develop competency within this, because if you as a teacher don’t have a good knowledge base regarding these questions, then it’s difficult to see the benefit of driving these issues forward. You would think that working with sustainability is obvious, but unfortunately this is not the case.” (IP11, Faculty board)*

#### Incentives for collaboration

Aside from overall professional development among teachers, participants described that working with integrating these perspectives had incentivised collaboration within and between universities. The need for exchanging ideas and expertise and gaining inspiration from other educational institutions was found to be particularly important when working with these topics which were considered relatively novel. Increased collaboration with universities abroad could compensate for expertise that may be missing at Swedish universities. The work with the 2030 Agenda had also contributed to incentives for collaboration across institutions, faculties and programmes within the universities through interprofessional learning.

*“During the development of the new curriculum we have initiated and developed collaboration with different universities, and I think we can use this in many different situations…we can help each other to develop this area, how we teach and how we examine. This is something I think we bring with us, that we collaborate across the country in a completely different way.” (IP6, PD)*

They also felt that it would be desirable if teachers could have access to a network focusing on the exchange of pedagogical methods and materials that they could use when developing teaching activities for their respective fields.

#### Global health as a selling point for incoming students

Participants argued that the reformation of medical education creates an opportunity to become a more attractive medical programme. One participant particularly emphasised that integrating global health and sustainable development could be a way of specialising their medical education so that students with specific interest in these questions would be incentivised to apply to their university.

*“We even discussed that we want to do this so well, that students who are interested in these issues will select this university knowing that they will get a well thought-out education within these area; that is our ambition.” (IP8, PD)*

#### Digitalisation brings global perspectives to the classroom

Collaboration and teacher exchanges between educational institutions in different countries were facilitated by new habits of using digital tools. Participants stated that increased digitalisation can bring global perspectives to the classroom in Sweden and highlight global health aspects in medical education without the need for geographic mobility.

*“It has become more evident that we need to be global on a local level. We can use digital solutions; we need to be more flexible in our way of thinking…that you don’t have to travel to achieve a global perspective - global perspectives are more than physical mobility.” (IP10, Faculty board)*

## Discussion

This study is the first to explore the perspectives of key stakeholders in medical education on the role of global health and sustainable development in Swedish medical education. Stakeholders discussed the challenges and opportunities associated with the modification of medical education, which was seen as necessary modernisation to fit the changing societal perception of the role of medical doctors. The participants emphasised a thorough anchoring process and ownership as key components for the implementation of these curriculum changes. However, finding conceptual consensus on what the terms “global health” and “sustainable development” mean was seen as a challenge, associated with resistance to the integration of these concepts. To overcome this, it was suggested that global health and sustainable development could be integrated with other components of the curricula. Moreover, participants felt that global and sustainability perspectives can contribute to an overall increase in the quality of the medical education and provide opportunities for collaboration and utilization of digital tools.

The findings indicate relative consensus amongst key stakeholders in Swedish medical education regarding the need for the inclusion of global health and sustainable development in the curriculum. However, there were disagreements among participants regarding how and to what extent such perspectives should be included. One reason for this was the differing views of global health and sustainable development, where some viewed this as a *“niche topic”*, relevant for one-two students per class who may choose a public health profession or have a special interest in the topics, whereas others described this as general knowledge that should be required for all medical doctors. The differing views appear to be intrinsically linked to different understandings of what the terms mean, a notion which has been similarly described previously amongst medical educators [21]. Similarly, the meaning of “global health” remains highly contested also among those active in the field [22], with more recent conceptualisations focusing on equity and health for all, rather than a specific geographic focus [6]. The ongoing debate regarding the meaning of global health and current shifts in attitudes on how to teach and practice global health [23, 24] may contribute to the conceptual uncertainty described by participants in this study. There was similar uncertainty regarding the meaning of “sustainable development”, which is a more recent term only rarely utilised in academic literature in relation to medical education.

According to the new Swedish medical programme, the medical schools have a responsibility to ensure that the students meet the learning outcomes corresponding to knowledge in global health and sustainable development which are mandatory components of the curriculum. However, who is de facto accountable for this implementation appears to be unclear, and participants had differing views about this. Implementation of these curriculum changes is likely to require different types of actions and engagement at different levels of the organisation, as the roles and responsibilities vary. For example, at the faculty level, global health and sustainable development were seen within the context of the university’s societal responsibility, and systems and processes such as financing and strategy were emphasised as keys for successful implementation. At the programme level, more emphasis was placed on the practical processes, including concretising the issues, setting realistic course and examination targets and building capacity among teachers. A central *“function”* was often requested, both from faculty- and programme-level members, to provide more clear directives on what *“global health”* and *“sustainable development”* mean and ensuring that they permeates the entire programme, faculty or, even, university. Yet, the question of whose responsibility this is remains unanswered.

A frequent concern raised was the challenge in anchoring this process throughout the entire organisation, including the students and teachers. Although student interest was broadly seen as high, resonating with literature from Sweden [16] and elsewhere [11] that has previously documented high student interest in the field, some participants noted that there appear to be student groups that do not share this interest. Students’ resistance to integration of global health and sustainable development in medical education has similarly been decribed at Harvard Medical School, where medical students interested in pursuing laboratory sciences or wanting to focus on the *“basic knowledge”* opposed the actions of making a course on global health and social medicine obligatory in the preclinical curriculum [25]. Active student involvement in the curriculum development processes was, surprisingly, emphasised to a lesser extent, which could be seen as a missed opportunity in the anchoring process, since student involvement in curriculum development has been linked to a feeling of ownership over their education [26]. Although engaged teachers and students were undeniably seen as important for the curriculum development relating to global health and sustainable development, it remains unclear how the programmes intend to utilise these groups to integrate these concepts in the organisation at large. Similarly, although recent literature has argued for the need of co-development of global health curricula through partnerships between institutions in high-income countries and low- and middle-income countries to ensure bidirectionality [27, 28], participants did not express an explicit need for external partnerships in the curriculum development process.

The findings that have emerged in this study indicate both challenges and opportunities associated with the processes of including global health and sustainable development in medical education. The role of global health and sustainable development in medical education remains a complicated issue, and the extent of their inclusion is still contested. The main challenge to operationalisation of the suggested curriculum changes appears to be uncertainties pertaining to ownership of these issues and their implementation and hence accountability. The lack of consensual understanding of these concepts in turn affects how they are prioritised and operationalised.

### Methodological considerations

This study used in-depth interview methods for data collection, a process that allowed the participants to express themselves freely. The detailed presentation of the research process increases the study’s transparency to other researchers. The use of an interview guide enhances dependability, and the analytical process, including consensus-building through feedback loops, strengthens the confirmability of the findings. The fact that two different interview guides were used for faculty board members and programme-level stakeholders can also be seen as a strength, since the questions were tailored to the specific positions. Finally, the inclusion of sustainable development in this study can be seen as a strength, since this is an important and timely perspective that is only rarely described in academic literature on medical education.

The study also has some limitations. Although data saturation was deemed to be satisfactory as nearly all eligible key stakeholders were included, it cannot be excluded that new information could have been obtained if further interviews had been conducted. Another possible limitation is that perspectives from students, who can arguably also be seen as “key stakeholders” in this field, are missing from the study. However, we believe that student perspectives should be further explored in a separate study, since they are seen as a separate set of “key stakeholders” compared with the group represented in this study. The fact that the interviewer in the study was a medical student could also be seen as a source of bias, potentially steering participants towards responses which they thought the student would want to hear. Yet, we believe that this risk was minor, as the student had no previous relationships with the participants, and refrained from sharing personal opinions. Arguably, interviews over Zoom can also be seen as suboptimal, with recent studies identifying disadvantages with the method, including the need to intentionally build rapport, technical issues, additional planning needs, privacy risks [29]. However, these risks were deemed to be insignificant in this study, since the topic was not sensitive. Although live-time video interview techniques have only recently become widespread and are still often seen as a *“second choice”* to the *“gold standard”* of face-to-face interviews, they have become widely accepted in academia and deemed to adequately resemble in-person interview situations [30]. Finally, it is unclear to what extent the results of this study are transferable to other contexts, where the medical education or the understanding of global health and sustainable development is different. However, other European studies have identified similar challenges relating to the integration of global health in medical education, suggesting that the results may be of relevance to other education systems in the region.

## Conclusion

Medical institutions must equip future doctors with the necessary skills and knowledge to effectively address the needs of a globalised society. This includes fostering an understanding of global health and sustainable development, enabling them to navigate the complex challenges and opportunities of our interconnected world. However, finding a shared understanding of global health and sustainable development is a challenge, which leads to resistance to the concepts due to fear of curriculum overload. To overcome this, strategic work to anchor the process of these curriculum changes is needed. Curriculum development can further be faciliated by bridging the gap to “clinical sciences” by integrating global health and sustainable development with other topics and developing existing components of the curriculum.

## Data Availability

Upon request, please contact corresponding author (LV).
